# Surface Mechanism of Fe^3+^ Ions on the Improvement of Fine Monazite Flotation With Octyl Hydroxamate as the Collector

**DOI:** 10.3389/fchem.2021.700347

**Published:** 2021-07-22

**Authors:** Qingzhu Zheng, Yunlou Qian, Dan Zou, Zhen Wang, Yang Bai, Haidong Dai

**Affiliations:** ^1^ Intelligent Safe Collaborative Innovation Center, Zhejiang College of Security Technology, Wenzhou, China; ^2^ Key Laboratory of Solid Waste Treatment and Resource Recycle Ministry of Education, Southwest University of Science and Technology, Mianyang, China; ^3^ Mechanical and Electronic Engineering Institute, Wenzhou University of Technology, Wenzhou, China

**Keywords:** monazite, fine particles, Fe^3+^, flotation, hydroxamate

## Abstract

Froth flotation of fine minerals has always been an important research direction in terms of theory and practice. In this paper, the effect and mechanism of Fe^3+^ on improving surface hydrophobicity and flotation of fine monazite using sodium octyl hydroxamate (SOH) as a collector were investigated through a series of laboratory tests and detection measurements including microflotation, fluorescence spectrum, zeta potential, and X-ray photoelectron spectroscopy (XPS). Flotation tests have shown that fine monazite particles (−26 + 15 μm) cannot be floated well with the SOH collector compared to the coarse fraction (−74 + 38 μm). However, adding a small amount of Fe^3+^ to the pulp before SOH can significantly improve the flotation of fine monazite. This is because the addition of Fe^3+^ promotes the adsorption of SOH and greatly improves the hydrophobicity of the monazite surface. This can result in the formation of a more uniform and dense hydrophobic adsorption layer, as shown by the fluorescence spectrum and zeta potential results. From the XPS results, Fe^3+^ reacts with surface O atoms on the surface of monazite to form a monazite–O_surf_–Fe group that acts as a new additional active site for SOH adsorption. A schematic model was also proposed to explain the mechanism of Fe^3+^ for improving surface hydrophobicity and flotation of fine monazite using octyl hydroxamate as a collector. The innovative point of this study is using a simple reagent scheme to float fine mineral particles rather than traditional complex processes.

## Introduction

Rare earth metals are an important input to the development and manufacture of many green and high-tech products. Rare earth elements (REEs) have important strategic value and are called “the mother of new materials’’ (Jordens et al., 2013). There is still little value in mining the world's rare earth deposits, and only a few deposits from China and the United States are doing large-scale mining. The ore types of these deposits are the main bastnaesite and monazite (Mancheri et al., 2019). Monazite [REPO_4_] mineral belongs to the monoclinic system and is one of the important minerals of mixed light rare earth resources in northern China (Yang et al., 2007). According to current research, flotation is the most effective way to concentrate monazite from related gangue minerals compared to the combined gravity–magnetic–electrostatic enrichment process (Zhang and Honaker, 2018). In this way, the difference in the floatability of monazite and the associated gangues could be enlarged and a basic condition for flotation separation could be created (Sis and Chander, 2013). Consequently, the selective and powerful collectors for the monazite flotation system are important research subjects of related researchers. Collecting ability is the most important index of a mineral collector. The more powerful the collecting ability of the collector is, the more hydrophobic the mineral surface it adsorbed would be, and the greater the sticking probability between the mineral and the bubble is (Yoon and Luttrell, 1989; Rosa and Rubio, 2018).

In the actual production process, oleic acid has long been the main collector of flotation of rare earth minerals. It works through chemisorption between –COO^−^ of a reagent molecule and the metal ion or metallic hydroxyl complex on the mineral/water interface. However, its selectivity is so low that the separation efficiency is low [6]. Hydroxamate is the significant alternative collector for monazite flotation and still under further study. It is an active reagent that reveals the properties of amides and oxime acids and is used for flotation of clay, copper oxide, cassiterite, etc. It can be adsorbed onto minerals by chemisorption between the metal ions on the mineral surface and the two O atoms in the molecule to form powerful five-membered ring chelation adsorption (Marion et al., 2013). Many studies have attempted to use hydroxamate as a collector for the typical rare earth mineral, bastnaesite, and have proven that hydroxamate is more effective than fatty acid collectors. In the monazite flotation, researchers are also beginning to test its efficiency. Pavez et al. found that using octyl hydroxamate as the collector could promote the flotation of monazite compared with sodium oleate (Pavez and Peres, 1994). Wang et al. studied the interaction mechanism of octyl hydroxamate on the monazite surface and showed that the octyl hydroxamate molecule could break through the electrostatic repulsion between the monazite surface and the reagent anions to form chemisorption, confirming the powerful adsorption performance (Wang et al., 2019).

However, recent laboratory experiments have shown that hydroxamate is less effective for the flotation of fine monazite particles. There are many ways to enhance fine mineral flotation, such as microbubble flotation, flocculation flotation, and carrier flotation (Farrokhpay et al., 2020). In this paper, we adopted another method by further improving the flotation of hydroxamate by adding Fe^3+^. The addition of Fe^3+^ increased the flotation recovery of monazite, and the mechanism was investigated by microflotation tests, surface micropolarity detection, zeta potential measurements, and X-ray photoelectron spectroscopy (XPS). The innovative point of this study is that instead of using complex processes (bubble flotation, agglomeration flotation, and carrier flotation), a simple reagent scheme (Fe^3+^ only added) is used to float fine mineral particles.

This study could serve as a reference for more effective flotation of fine-grained ores, as the world faces the challenge of dealing with low-grade, fine-disseminated ores (Ignatkina et al., 2019; Jin et al., 2021).

## Materials and Methods

### Reagents and Mineral Samples

**FIGURE 1:**
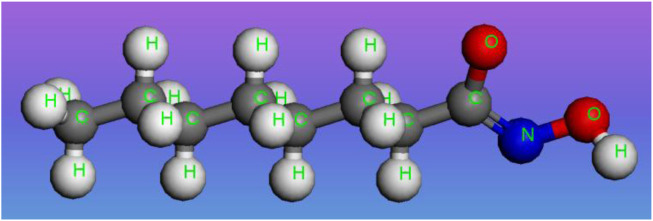
Molecule structure of octyl hydroxamate (white, H; gray, C; red, O; blue, N).

Sodium octyl hydroxamate (SOH) with a purity of 98% was used as a collector in flotation experiments. Analytical grade ferric trichloride (FeCl_3_) was used as a surface modifier in flotation experiments. Both SOH and FeCl_3_ were purchased from Sinopharm Chemical Reagent Co., Ltd., China. The chemical structure of sodium octyl hydroxamate is shown in Figure 1, which was drawn and optimized using the Materials Studio 5.0 package. The pH was regulated with sodium hydroxide (NaOH) and hydrochloric acid (HCl) stock solutions (also from Sinopharm Chemical Reagent Co., Ltd.). The water used in all the tests was deionized water with a resistivity of about 18.2 MΩ cm.

**FIGURE 2:**
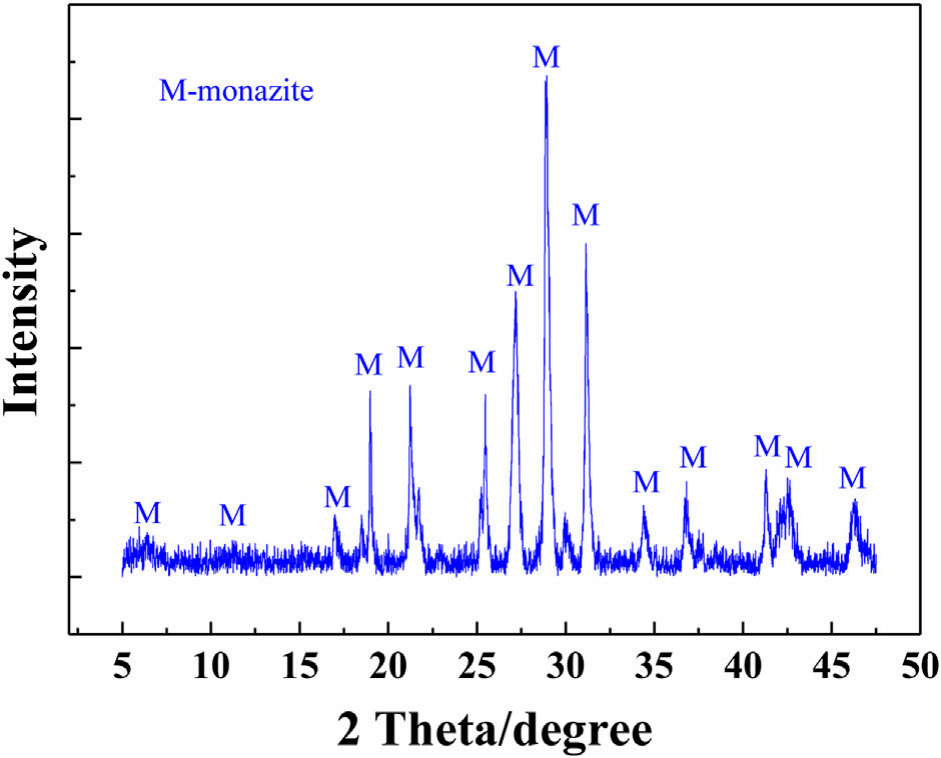
XRD patterns of monazite samples with monazite phase labeled.

Massive monazite crystals were selected from a rare earth mine in Guangxi Zhuang Autonomous Region, China. They were further purified through repeated gravity concentration using the table concentrator. Then, the purified samples were ground in the ceramic ball mill and screened to obtain different fractions (−26 + 15 μm, −74 + 38 μm, and −15 μm). The weight contents of REO and P_2_O_5_ in the purified monazite samples shown in Table 1 are 60.15 and 24.81%, respectively. The approximate theoretical grade of monazite mineral, representing its purity, was about 95%. The XRD spectrum was also obtained and is shown in Figure 2 with the monazite phase labeled on the corresponding peaks, by which the high purity of the purified samples was confirmed again.

**TABLE 1:** Chemical analysis results of the purified monazite.

Element content (wt. %)								
Mineral	TiO_2_	REO	ZrO_2_	SiO_2_	ThO_2_	Fe_2_O_3_	P_2_O_5_	Al_2_O_3_
Monazite	0.96	60.15	0.08	2.19	5.67	0.39	24.81	0.41

### Microflotation

Microflotation tests were performed in a hanging trough flotation cell (XFG, 40 mlcell, Jilin Exploration Machinery Plant, China), the impeller speed set as 1990 rpm. 2.0 g (±0.002 g), and the mineral samples were placed in the 40 mlcell with 35 ml of deionized water. After agitating for 2 min, the pH regulator was added. The desired pH value was determined using a pH meter (PHS-3C), followed by conditioning the pulp for another 2 min. Then, the promoter (if needed) and collector were added successively; the conditioning times for the promoter and collector were 2 and 3 min, respectively. Both the floated and non-floated products were filtered, dried, and then weighed to calculate flotation recovery. Previous studies have reported a similar experimental flow chart with the same flotation machine (Qian et al., 2020). Three flotation tests were performed under the same conditions, and the average values were reported.

### Fluorescence Emission Spectroscopy

**FIGURE 3:**
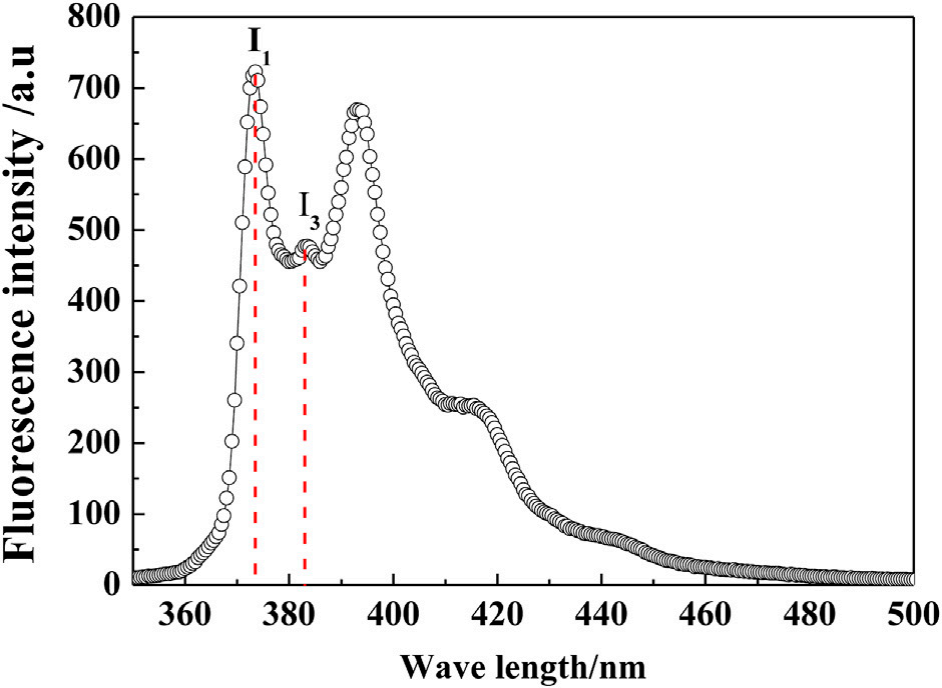
Fluorescence spectrum of pyrene in distilled water.

Pyrene was dissolved into hot water until saturation to prepare the stock solution, then cooled to 25°C, and filtered. The concentration of pyrene in the solution was determined to be 6.53 × 10^−7^ mol/ L. Then, the monazite samples for fluorescence emission spectroscopy measurements were prepared by mixing a pyrene stock solution with a reagent and mineral pulp and allowing it to stand for 1.5 h to equilibrate. The pyrene steady-state emission spectra were obtained by using a Hitachi F-4500 fluorescence spectrophotometer. There are five characteristic peaks in the fluorescence spectrum of pyrene in distilled water (Figure 3). The ratio between the intensity of the first and third peaks of pyrene, I_1_/I_3_, is sensitive to the polarity of the medium located by the probe molecules. It has been revealed to change with the polarity of the solvent, of which the values are <1.0 in the nonpolar solvent, 1–1.2 in surfactant micelles, and 1.6–2 in water (Wang et al., 2015).

### Zeta Potential Measurements

The zeta potential of minerals before and after treatment with reagent(s) was measured using a Coulter DELSA 440S II electrokinetic instrument. A powder sample (−15 μm, 2.0 mg) was first ground to ∼2 μm in an agate mortar and then transferred to a 100 ml beaker, containing 50 ml of 1 × 10^−3^ mol/ L KCl background electrolyte, at a given pH and reagent concentration. After being magnetically stirred for 10 min followed by settling for 7 min, the supernatant liquor was used for zeta potential measurements. Repeated tests revealed a measurement error of ± 2 mV at 25.0 ± 0.5°C.

### XPS Detection

The XPS spectra for monazite particles with and without treatment by reagent(s) with the same concentration as in flotation were recorded with a K-Alpha 1063 (Thermo Scientific Co., United States) spectrometer, which employs Al Kα as a sputtering source at 12 kV and 6 mA with 1.0 × 10^−9^ Pa pressure in the analytical chamber. The C 1s peak served as a reference to binding energy (BE) for uncharged hydrocarbon at 284.8 eV, and the BE in all other spectra for that sample corrected for this shift. The quantification and curve fitting of the spectra were determined using XPSPEAK 4.1 software.

## Results and Discussion

### Microflotation

**FIGURE 4:**
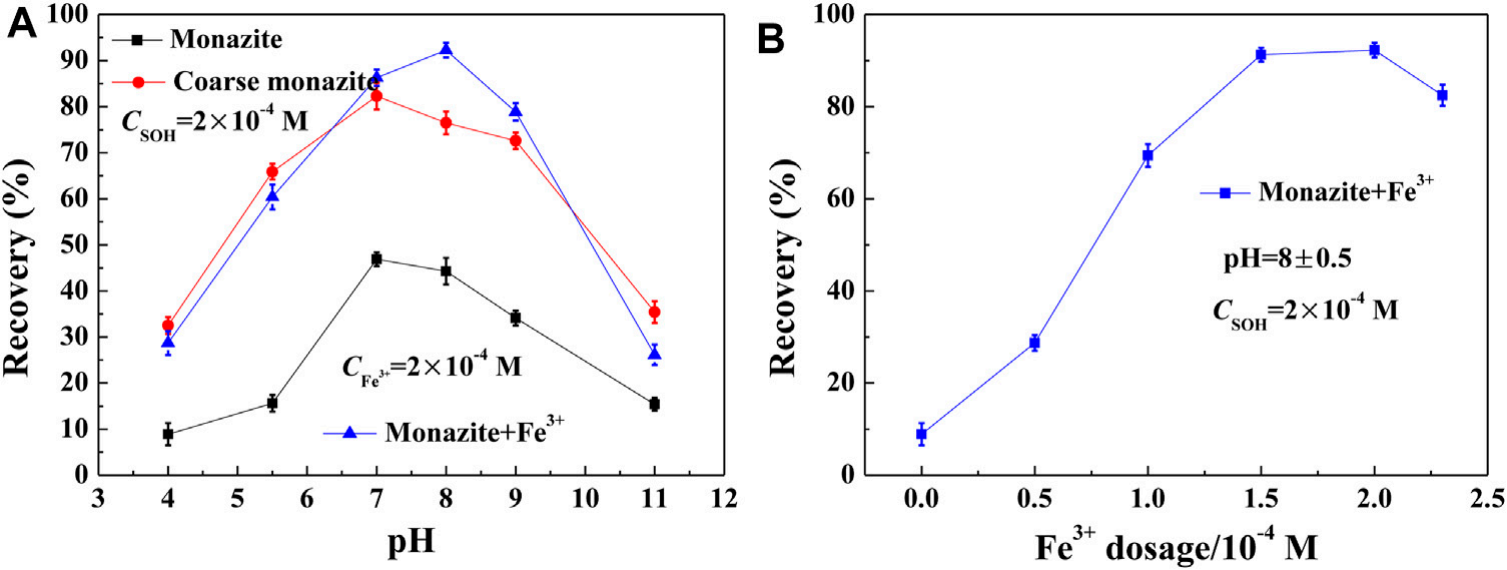
Monazite recovery with SOH as a function of **(A)** pH and **(B)** Fe^3+^ dosage. (Coarse monazite refers to –74 + 38 μm fractions, while monazite represents –26 + 15 μm fractions.)

In order to determine the flotation response of monazite particles (−26 + 15 μm) with or without the addition of Fe^3+^, and the difference in the flotation behavior compared with the coarser (−74 + 38 μm) ones, microflotation tests were conducted with 2 × 10^−4^ M SOH as the collector. The results corresponding to the influence of pH and Fe^3+^ dosage on monazite recovery are shown in Figures 4A,B, respectively.

As shown in Figure 4A, the flotation recovery rate of monazite increases with the increasing pH from 4 to 7 and decreases with the increasing pH if Fe^3+^ was not added to the pulp. Flotation of coarse monazite particles behaves similarly, with an optimal recovery of 82.3% at pH 7, but the corresponding value of the monazite fraction −26 + 15 μm is only 46.9%. The effect of pH on mineral recovery is due to the low degree of dissociation of SOH to SO^−^ in a low pH range, the active constituent to adsorb on the monazite surface, and the formation of hydrophilic metal hydroxide on the monazite surface in a high pH range. It suggests that the flotation recovery rate of fine monazite particles is low compared to that of the coarse one. Some studies on fine particles (below −38 μm) indicate that the adsorption of the collector with the mineral surface is insufficient and uneven due to most of the surface atoms having no activity for the collector (Karakas and Hassas, 2016). In this paper, we investigated the promotion of flotation of fine minerals using metal ions. The optimal flotation recovery (92.3%) was achieved at pH 8 in the presence of 2 × 10^-4^ M Fe^3+^ in the pulp. These results clearly show the improvement in Fe^3+^ in the flotation of fine monazite using SOH as a collector.

Figure 4B displays the monazite recovery with SOH as a function of Fe^3+^ dosage.

Increasing the Fe^3+^ dose rapidly increased the flotation recovery rate of monazite from 8.9% at 0 M to 91.3% at 1.5 × 10^−4^ M, which then remained unchanged with the further increase in dose. Continuing to increase the addition of Fe^3+^, the flotation recovery begins to decrease, being attributed to the mass production of Fe(OH)_3_ precipitates at high Fe^3+^ dosage, which inhibits the flotation of monazite mineral particles (Peng et al., 2017). The addition of 1.5 × 10^−4^ M Fe^3+^ greatly improved the flotation ability of 2 × 10^−4^ M SOH on the difficult floated monazite fractions (−26 + 15 μm).

### Surface Micropolarity Detection

**FIGURE 5:**
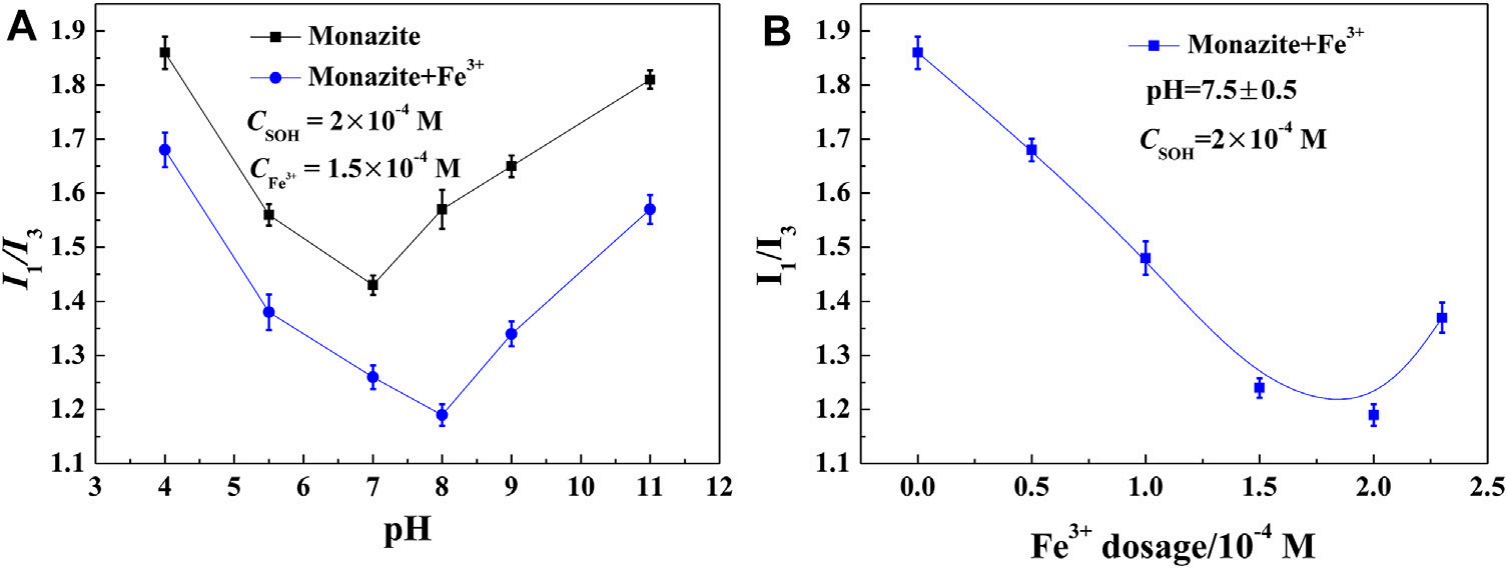
I_1_/I_3_ value of the monazite surface treated with SOH as a function of **(A)** pH and **(B)** Fe^3+^ dosage.

In general, floating minerals have a more hydrophobic surface, which makes them easier to attach to bubbles and lift to the pulp surface. For mineral surfaces, the weaker the polarity, the stronger the hydrophobicity (Liu et al., 2017; Liu et al., 2017). The pyrene fluorescence test has been proven to be an effective technique to obtain structural information of the adsorbed layer on minerals (Chandar et al., 1987). Here, it was adopted to characterize the polarity/hydrophobicity of the monazite surface in different reagent conditions, and the results are shown in Figures 5A,B. The lower the I_1_/I_3_ value of the mineral pulp, the more hydrophobic the mineral/water interface. As shown in Figure 5A, in the absence of Fe^3+^, the I_1_/I_3_ value of the monazite surface decreases first and then increases, and the lowest I_1_/I_3_ value occurs at pH 7. In contrast, in the presence of Fe^3+^, the I_1_/I_3_ value changes in the same way as in the absence of Fe^3+^, with the lowest I_1_/I_3_ value occurring at pH 8. These findings show that the most hydrophobic monazite surface could be prepared at pH 7 without Fe^3+^ and at pH 8 with 1.5 × 10^−4^ M Fe^3+^, which is consistent with the best flotation recovery of monazite in corresponding conditions. At lower pH 4 or higher pH 11, almost all the I_1_/I_3_ values lie in the 1.2–1.6 range for water, presenting a stronger hydrophilic monazite surface at these pH values; hence, monazite recovery is poor. At pH 7 without Fe^3+^, the I_1_/I_3_ value is 1.43, indicating that the monazite surface is still somewhat hydrophilic, as there is not too much SOH adsorbed on the mineral surface. For the case with the Fe^3+^ addition, the I_1_/I_3_ values are lower than 1.4 in the pH range of 5.5–9, meaning that the hydrophobicity is high, and the SOH anions may be adsorbed by semi-micelle adsorption (Zhang et al., 2007).

Comparing the I_1_/I_3_ values of monazite pulp with and without the addition of 1.5 × 10^−4^ M Fe^3+^, Fe^3+^ may improve the surface hydrophobicity of monazite over the entire pH range. Continuing the observation, the variable I_1_/I_3_ values after the addition of Fe^3+^ are maximal at pH 8. This is in good agreement with the largest change in the monazite flotation recovery rate. The influence of Fe^3+^ dosage is also investigated in the results shown in Figure 5B. The variation tendency also coincided with the flotation behavior: with 1.5 × 10^−4^ M SOH only in the pulp, the mineral surface is very hydrophilic (>1.85 I_1_/I_3_ value); as the Fe^3+^ dosage increases, the most hydrophobic monazite particles are obtained at 1.5–2 × 10^−4^ M Fe^3+^. All these results explain the improvement in Fe^3+^ addition in monazite flotation with collector SOH in terms of mineral polarity/hydrophobicity.

### Zeta Potential Measurements

**FIGURE 6:**
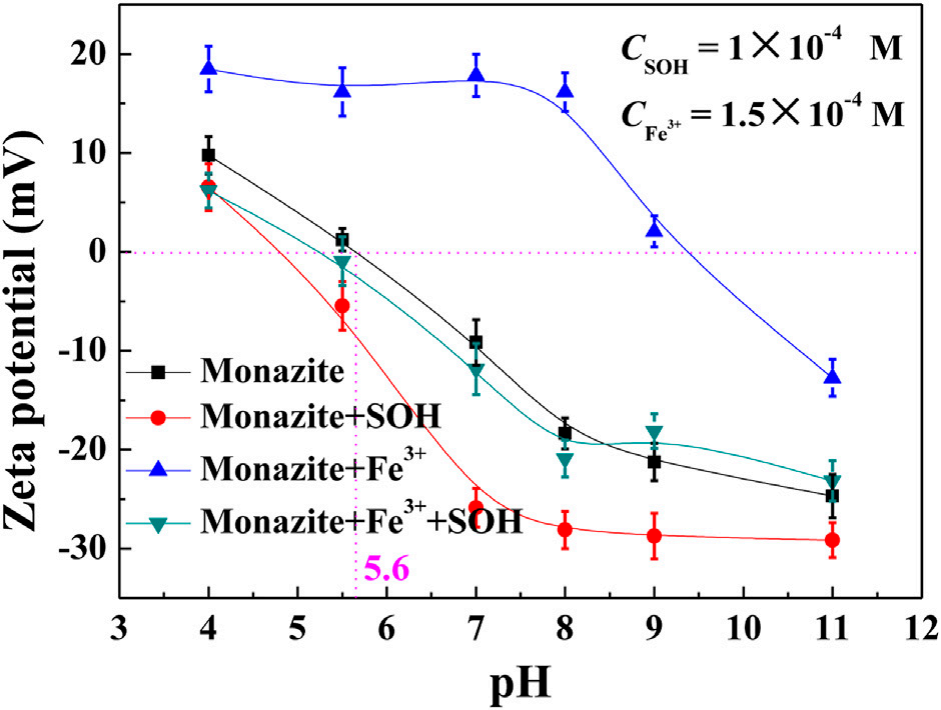
Zeta potential of monazite under different reagent conditions as a function of pH.

For the flotation system, the adsorption of heteropolar collector molecules onto the mineral surface induces the hydrophobicity and flotation of mineral particles. In this paper, SOH is an anionic collector, while Fe^3+^ is cationic, so the adsorption of them does result in the change in the zeta potential of the monazite surface. Hence, the zeta potential of the monazite surface was measured under different reagent conditions to delineate interfacial interaction phenomena, and the results are presented in Figure 6. The isoelectric point (IEP) of raw monazite in the background electrolyte solution is 5.6, which is close to the value 6.0 or 5.3 tested by Zhang (2017) and Nduwa Mushidi (2016), respectively. The zeta potential gradually decreases with the increasing pH.

When 2 × 10^−4^ M collector SOH was added into the solution, the zeta potential of the monazite surface became more negative in the whole pH range. The variation is slight as shown in Figure 6, illustrating that a small amount of SOH was adsorbed onto monazite. The greatest change (∼14 mV) in the zeta potential between monazite and monazite/SOH systems occurs at pH 7. In the pH range 5.6–11, the monazite surface is negatively charged, but the negatively charged SOH anions can still be adsorbed to make the zeta potential of the monazite surface more negative, indicating chemisorption between SOH and the monazite surface (Gao Y. et al., 2018). Simply adding 1.5 × 10^-4^ M Fe^3+^ will significantly increase the zeta potential and shift the IEP to about 9.4, which is ascribed to the electrostatic attraction between the negatively charged monazite surface and cationic Fe^3+^ ions. The greatest change (∼34 mV) in the zeta potential between monazite and monazite/Fe^3+^ systems occurs at pH 8.

The more important phenomenon is that, with SOH addition after Fe^3+^, the greatest change (∼36 mV) in the zeta potential between monazite/Fe^3+^ and monazite/Fe^3+^/SOH systems also occurs at pH 8; the zeta potential change between monazite/Fe^3+^ and monazite/Fe^3+^/SOH systems is much larger than that between monazite and monazite/SOH systems. This suggests that the addition of Fe^3+^ can greatly promote the adsorption of SOH onto the monazite surface. The change in the optimal pH of monazite flotation from 7 to 8 is easy to understand based on the above analysis.

### XPS Measurements

**FIGURE 7:**
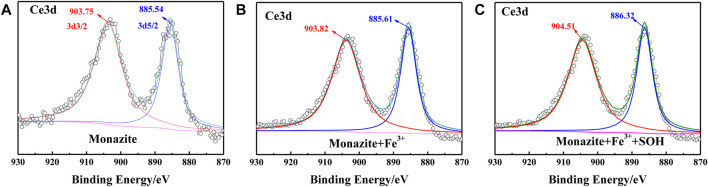
Ce3d XPS spectrum on the **(A)** pure monazite surface, **(B)** monazite surface treated with Fe^3+^, and **(C)** monazite surface treated with Fe^3+^ and SOH, respectively.

**FIGURE 8:**
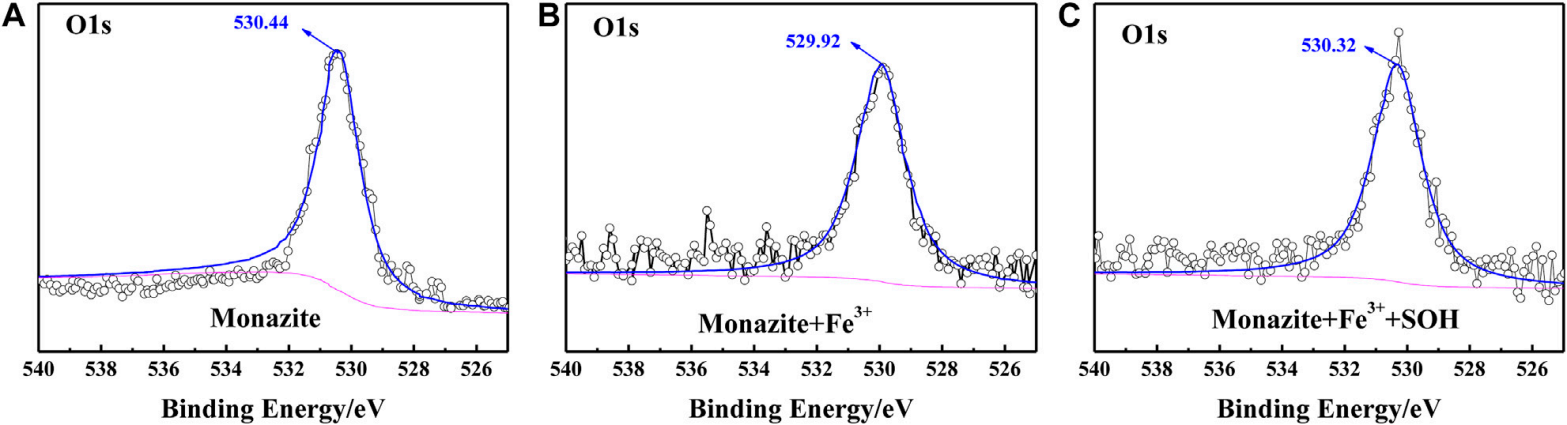
O1s XPS spectrum on the **(A)** pure monazite surface, **(B)** monazite surface treated with Fe^3+^, and **(C)** monazite surface treated with Fe^3+^ and SOH, respectively.

**FIGURE 9:**
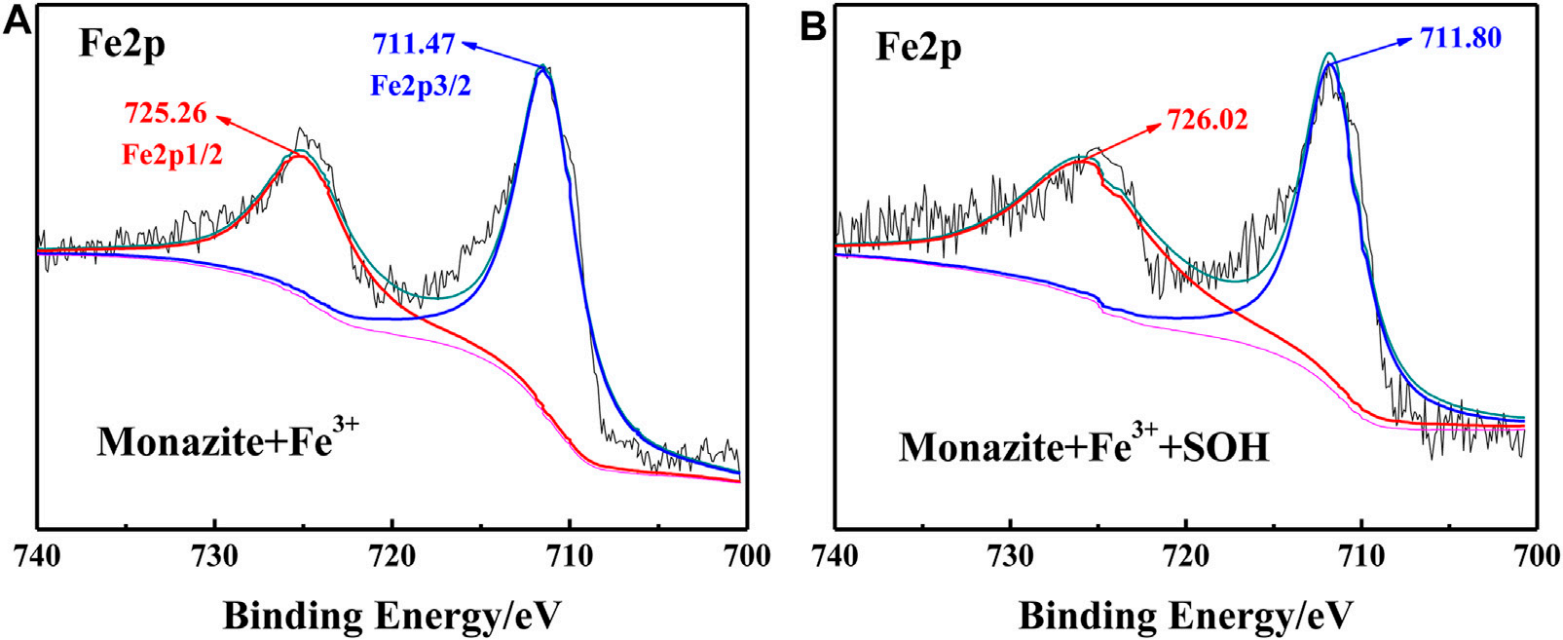
Fe2p XPS spectrum on **(A)** monazite surface treated with Fe^3+^ and **(B)** monazite surface treated with Fe^3+^ and SOH, respectively.

The adsorption of SOH on the monazite surface can be greatly improved by adding an adequate dose of Fe^3+^, thus increasing the surface hydrophobicity and mineral floatability. This has been proved by the results of microflotation, surface micropolarity detection, and zeta potential measurements. However, the mechanism of Fe^3+^ improving SOH adsorption on the monazite surface still needs further study. Here, the XPS tests were conducted for monazite before and after treatment with flotation reagent(s) because it is a proven means to analyze the interface interaction mechanism in the mineral flotation system (Zhang et al., 2019). The narrow-scan spectra corresponding to Ce3d, O1s, and Fe2p on the monazite surface in different conditions are shown in Figures 7–9, respectively.

Ce is the major REE on the surface of monazite and serves as the active site for adsorption of hydroxamic acid type reagents (Zhang and Honaker, 2017). As shown in Figure 7A, on the monazite surface without treatment with reagents, the XPS spectrum of Ce3d displays two peaks at 903.65 eV (3d3/2) and 885.46 eV (3d5/2) BE, ascribed to the Ce^3+^ ions in the monazite lattice (Espiritu et al., 2018). The peak shifts of Ce3d3/2 and Ce3d5/2 are +0.07 eV and +0.07 eV when Fe^3+^ was introduced into the pulp (Figure 7B). According to literature studies, if there is chemical bonding, an obvious shift of the XPS peak would be found (Sekine et al., 1996). Now, no obvious shift can be found after Fe^3+^ addition, which indicates there is no reaction between Fe^3+^ and the Ce atom on the monazite surface. When monazite was treated with both Fe^3+^ and SOH, the corresponding peak shifts relative to the mineral surface treated with Fe^3+^ alone are +0.69 eV for Ce3d3/2 and +0.71 eV for Ce3d5/2, respectively (Figure 7C), which are all obviously greater than the resolution of the XPS instrument 0.2 eV, indicating chemical bonding between SOH and the Ce atom on monazite.

The O1s spectrum was obtained with the peak at the binding energy of about 531 eV (Figure 8). This BE value has been regarded as corresponding to bridging oxygen atoms (Figure 11) of the monazite surface. During hydroxylation of the monazite surface, these bridging oxygen atoms may form oxhydryl (monazite–O_surf_–H) on the mineral surface (Tian et al., 2018). When the monazite surface was treated with Fe^3+^, a −0.52 eV shift from 530.44 to 529.92 eV can be found by comparing the corresponding spectra (Figure 8B), which suggests a chemical interaction of Fe^3+^ with the hydration hydroxyl on the monazite surface. With both Fe^3+^ and SOH treatments, the peak in the O1s spectrum shifts by +0.4 eV to 320.32 eV (Figure 8C) in comparison with the O1s spectrum of monazite treated with Fe^3+^ alone. This may illustrate chemical bonding between the monazite–O_surf_–Fe group and the SOH anions, but it is difficult to determine because there are also O atoms in the SOH molecule.

The Fe2p spectra treated by Fe^3+^ alone and Fe^3+^/SOH-treated monazite surface are shown in Figures 9A,B, respectively. With only Fe^3+^ treatment, the peaks for Fe2p1/2 and Fe2p3/2 locate at 725.26 and 711.47 eV, respectively. In the Fe2p spectrum of monazite after treatment with Fe^3+^ and SOH, both the Fe2p1/2 and Fe2p3/2 characteristic peaks shift to higher BE values, locating at 726.02 and 711.80 eV, respectively. The chemical shifts +0.76 eV and +0.33 eV all suggest an interaction between the monazite–O_surf_–Fe group and the SOH anions does occur.

The atom content changes of the Ce, C, Fe, and N elements on the monazite surface were also calculated, and the consequences are shown in Table 2. After treatment with Fe^3+^, the Fe element (10.68%) occurs at the mineral surface, which also results in the decline of Ce content. When it was treated with SOH only, the C content increases to 15.88%, and the N element (6.09%) is detected on the monazite surface, indicating the adsorption of collector SOH. This also results in the decrease in Ce content on the monazite surface because the SOH anions mainly adsorb on the Ce active sites of the mineral surface, rendering the Ce atom being masked. For the monazite surface treated with both Fe^3+^ and SOH, the C and N contents increase sharply compared to those after treatment with only the SOH collector, also suggesting an improvement of the adsorption of SOH onto the monazite surface through the addition of Fe^3+^. This accompanies only a small decrease in the Ce content, meaning that Fe^3+^ addition may not mainly improve the adsorption of SOH on Ce active sites, but on the newly formed Fe active sites, reflected by the greatly declined Fe content (masked by the adsorbed SOH).

**TABLE 2:** Atomic proportions (at. %) of Ce, Fe, and N elements on the monazite surface.

Samples	Elements			
	Ce	C	Fe	N
Raw monazite	24.66	7.46	—	—
Fe^3+^-treated monazite	19.68	7.09	10.68	—
SOH-treated monazite	12.36	15.88	—	6.09
Fe^3+^/SOH-treated monazite	10.14	29.24	4.62	14.43

### Proposed Mechanism

**FIGURE 10:**
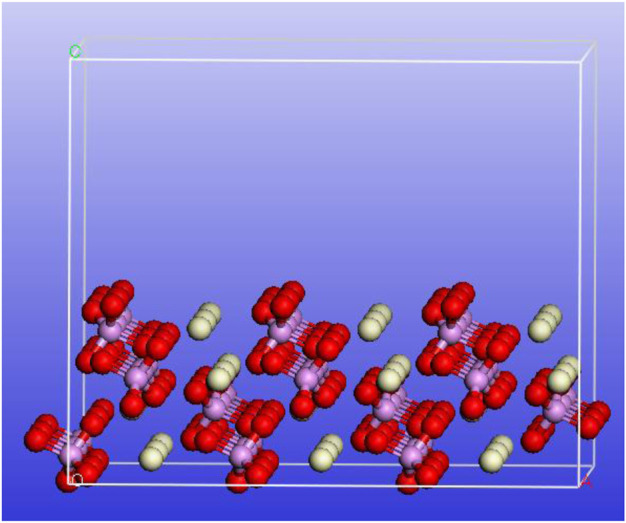
Schematic diagram of the 3 × 3 supercell of the monazite crystal with the (100) cleavage (top view) being cut.

The monazite (100) crystallographic plane is the distinct cleavage, and it would be exposed in large probability during crushing and grinding, so it is the main adsorbed object of flotation reagents (Montel et al., 1996). A 3 × 3 supercell of the monazite crystal was constructed with the (100) cleavage being cut (top view) using the Materials Studio 5.0 package. As shown in Figure 10, the phosphate radical and Ce atom arrange alternately. Due to the surface atoms having the additional dangling bonds, they are commonly active (Gao Z. et al., 2018).

**FIGURE 11:**
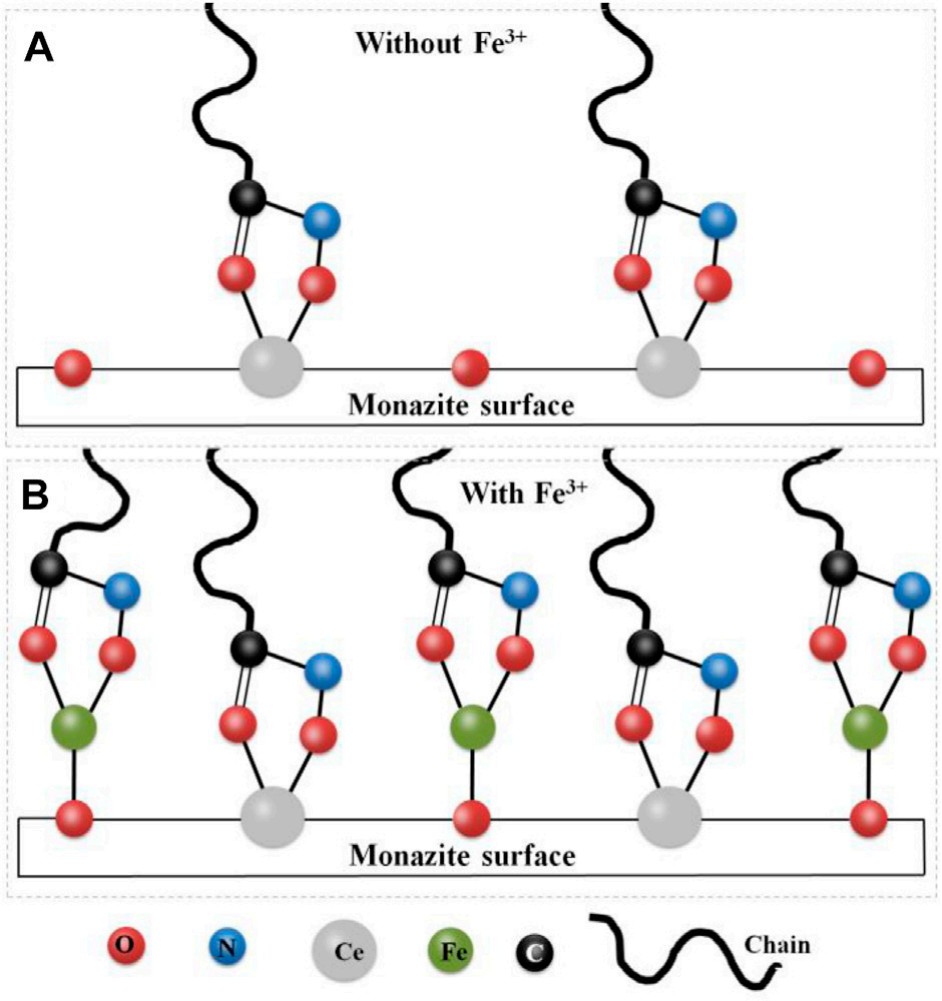
Schematic diagram of the improving mechanism of Fe^3+^ on the adsorption of SOH on fine monazite flotation: **(A)** without Fe^3+^ ions; **(B)** with Fe^3+^ ions.

In this paper, due to the absence of Fe^3+^ in the pulp, the SOH collector adsorbs onto the monazite surface primarily through chelation between the two O atoms of the molecule and the Ce active site. However, due to the fine size of the monazite particles (−26 μm), the adsorption of the collector is uneven and most of the Ce atoms are not present as active sites. The lower adsorption of SOH onto the monazite surface causes lower hydrophobicity, lower zeta potential change, lower N atom content change, and lower flotation recovery of monazite with only the SOH collector. The low hydrophobicity of the monazite surface renders it hard to adhere to the hydrophobic bubbles and be lifted onto the pulp surface (Verrelli et al., 2011; Ralston et al., 1999). With Fe^3+^ addition into the system, it reacts with the active surface O atom to form monazite–O_surf_–Fe groups, which are the newly formed active sites for SOH chelating adsorption. From the results of surface micropolarity detection, zeta potential measurement, and XPS analysis, the greater adsorption of SOH with the monazite surface can be observed after Fe^3+^ is being added before the addition of SOH, which would result in a dense SOH hydrophobic layer on the monazite surface. Hence, in the tests, the recovery of monazite flotation was improved because of the addition of Fe^3+^. Figure 11 is drawn to understand the mechanism of improvement of Fe^3+^ in adsorption of SOH to fine monazite and flotation.

## Conclusion

The study investigated the effects and mechanism of Fe^3+^ on improving surface hydrophobicity and flotation of fine monazite with octyl hydroxamate as the collector. The flotation results showed that fine monazite particles (−26 + 15 μm) could not sufficiently float using the SOH collector compared to the coarser fraction (−74 + 38 μm). By adding Fe^3+^ to the pulp before SOH, the flotation performance of SOH for fine monazite can be significantly improved at moderate pH and reagent doses. The added Fe^3+^ reacts with the surface O atom on the cleavage of monazite to form the monazite–O_surf_–Fe group. Chelation of two O and Fe atoms also functions as a new active site for SOH adsorption. Both the Fe and Ce active sites on the monazite surface can adsorb SOH anions, facilitating the adsorption of SOH on the monazite surface, possibly resulting in a more uniform and dense SOH hydrophobic adsorption layer. This is the reason for the improved flotation behavior.

## Data Availability

The raw data supporting the conclusion of this article will be made available by the authors, without undue reservation.
